# Mixed Reality-Based Navigation for Pedicle Screw Placement: A Preliminary Study Using a 3D-Printed Spine Model

**DOI:** 10.7759/cureus.59240

**Published:** 2024-04-28

**Authors:** Masayuki Ohashi, Masayuki Sato, Hideki Tashi, Keitaro Minato, Tatsuo Makino, Hiroyuki Kawashima

**Affiliations:** 1 Department of Orthopedic Surgery, Niigata University Graduate School of Medical and Dental Sciences, Niigata, JPN

**Keywords:** navigation, spine surgery, mixed reality, extended reality, healthcare tech contest

## Abstract

Background and objectives

Mixed reality (MR) is one of the image processing technologies that allows the user to manipulate three-dimensional (3D) virtual images (hologram). The aim of this study was to evaluate the accuracy of MR-based pedicle screw (PS) placement using 3D spine models.

Materials and methods

Using the preoperative CT data of a patient with adolescent idiopathic scoliosis (AIS) who had undergone posterior spinal fusion in our hospital, a 3D-printed spine model was created. On the other hand, a 3D hologram of the same patient was automatically created using the preoperative CT data uploaded to the Holoeyes MD service website (Holoeyes Inc., Tokyo, Japan). Using a Magic Leap One® headset (Magic Leap Inc., Plantation, FL), the 3D hologram with lines of predetermined PS trajectories was superimposed onto the 3D-printed spine model and PS were inserted bilaterally along with the trajectory lines from T5 to L3. As a control, we used a readymade 3D spine model of AIS and inserted PS bilaterally with a freehand technique from T4 to L3. The rate of pedicle violation was compared between the MR-based and freehand techniques.

Results

A total of 22 and 24 PS were placed into the 3D-printed spine model of our patient and the readymade 3D spine model, respectively. The rate of pedicle violation was 4.5% (1/22 screws) in the MR-based technique and 29.2% (7/24 screws) in the freehand technique (P = 0.049).

Conclusions

We demonstrated a significantly lower rate of PS misplacement in the MR-based technique than in the freehand technique. Therefore, an MR-assisted system is a promising tool for PS placement in terms of feasibility, safety, and accuracy, warranting further studies including cadaveric and clinical studies.

## Introduction

In modern spine surgery, the pedicle screw (PS) is one of the essential tools for spinal deformity correction due to its great three-column control. However, a misplaced PS can cause neural, vascular, or visceral injury because neurovascular and visceral structures are in close proximity to the pedicle and vertebral body. Therefore, to improve the accuracy of PS insertion, various screw insertion techniques have been introduced including freehand, fluoroscopic-guided, navigation-assisted, and robotic-assisted methods. Of these, image-guided navigation systems and robotic-assisted methods have been reported to provide higher accuracy in PS placement than the freehand technique and use of fluoroscopy [1-6]. However, advanced operative technology is generally an expensive investment, especially for the software and maintenance of the equipment [7]. Additionally, some navigation systems can increase surgical time [8] and radiation exposure for both patients and the surgical team [9].

Extended reality is an image-processing technology, which includes all immersive technologies, such as virtual reality (VR), augmented reality (AR), and mixed reality (MR) [10]. VR is a computer-generated simulation that excludes the real-world environment and visually immerses the user in a completely artificial three-dimensional (3-D) visual or other sensory environment. On the other hand, AR technology enables the overlap of a computer-generated virtual image with objects in the real environment, although the user cannot interact with the virtual image. Furthermore, MR can be conceptually seen as an extension of AR technology [11], allowing users to interact with and manipulate 3D data packets in both real and virtual environments. These technologies have been utilized in several medical fields, especially in surgery, where the user has a direct view of the surgical field with superimposed virtual images that provide anatomical information [12]. Nowadays, one of the most popular surgical fields where AR or MR are used is PS placement. Recent systematic reviews report an accuracy range of 1-5 mm for AR systems [13] and a mean clinical accuracy of 93.1% (range, 26.3-100%) in AR-assisted PS placement [14]. However, the majority of navigation systems based on AR require intraoperative computed tomography (CT) or 3D fluoroscopy, which is generally expensive and not available anywhere or anytime. In addition, intraoperative imaging increases radiation exposure and prolongs operative time. Therefore, as another option for navigation systems of PS placement, we have been developing a novel method based on MR, which allows the user to manipulate 3D virtual images. We aimed to evaluate the accuracy of MR-assisted PS placement without intraoperative imaging using a 3D-printed spine model of a patient with adolescent idiopathic scoliosis (AIS).

## Materials and methods

Clinical data of the patient with AIS

**Figure 1:**
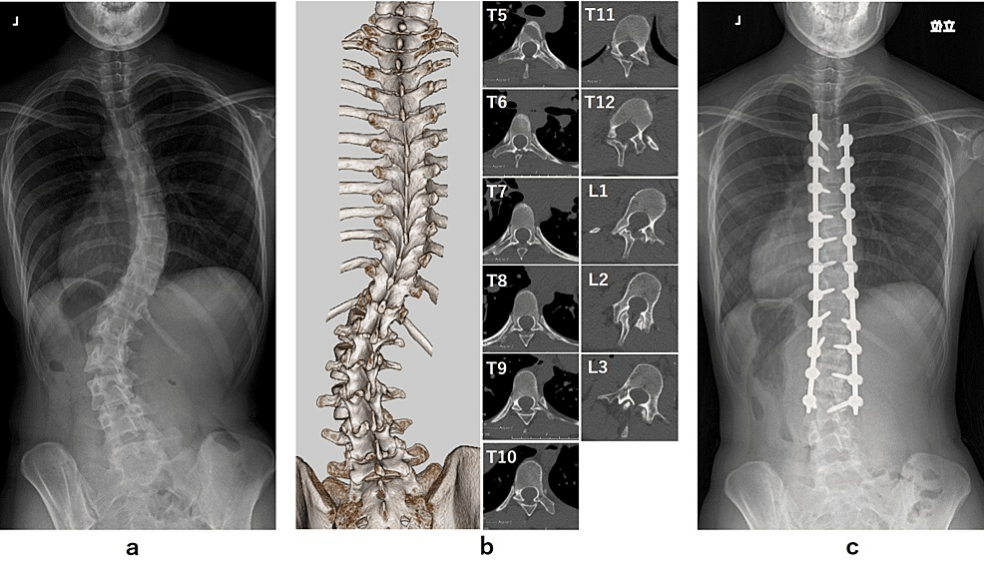
The imaging data of the patients with adolescent idiopathic scoliosis. a. Preoperative standing radiograph b. Preoperative computed tomography c. Postoperative standing radiograph

The patient is an 18-year-old female with AIS. Preoperative standing radiographs revealed a 48° main thoracic curve (T5-12) and a 50° lumbar curve (T12-L4) (Figure 1a). Plain T1-to-sacrum CT scans were taken preoperatively to obtain data according to the Digital Imaging and Communications in Medicine (DICOM) standards for the navigation system (Figure 1b). CT was performed using a multislice CT scanner (Philips Ingenuity Elite, Philips Healthcare, Amsterdam, Netherlands) with the following scanning parameters: tube voltage, 120 kV; modulated tube current achieved by automatic exposure control (Dose Right Index, Philips Healthcare, Amsterdam, Netherlands); rotation time, 0.5 s; pitch, 0.798; detector collimation, 64 sliced × 0.625 mm. She had undergone a posterior spinal fusion (PSF) with all PS constructs from T5 to L3 (Figure 1c). During surgery, PSs were inserted bilaterally from T5 to L3 under a frameless stereotactic image-guidance system (Stealth-Station 7, Medtronic, Sofamor Danek, Memphis, TN), in which preoperative CT data were used. We confirmed that all PSs were contained within the pedicle using postoperative CT except for two PSs with minimal lateral breach (<2 mm, in-out-in trajectory) at right T5 and T6.

Technical setup and PS insertion under MR guidance

**Figure 2:**
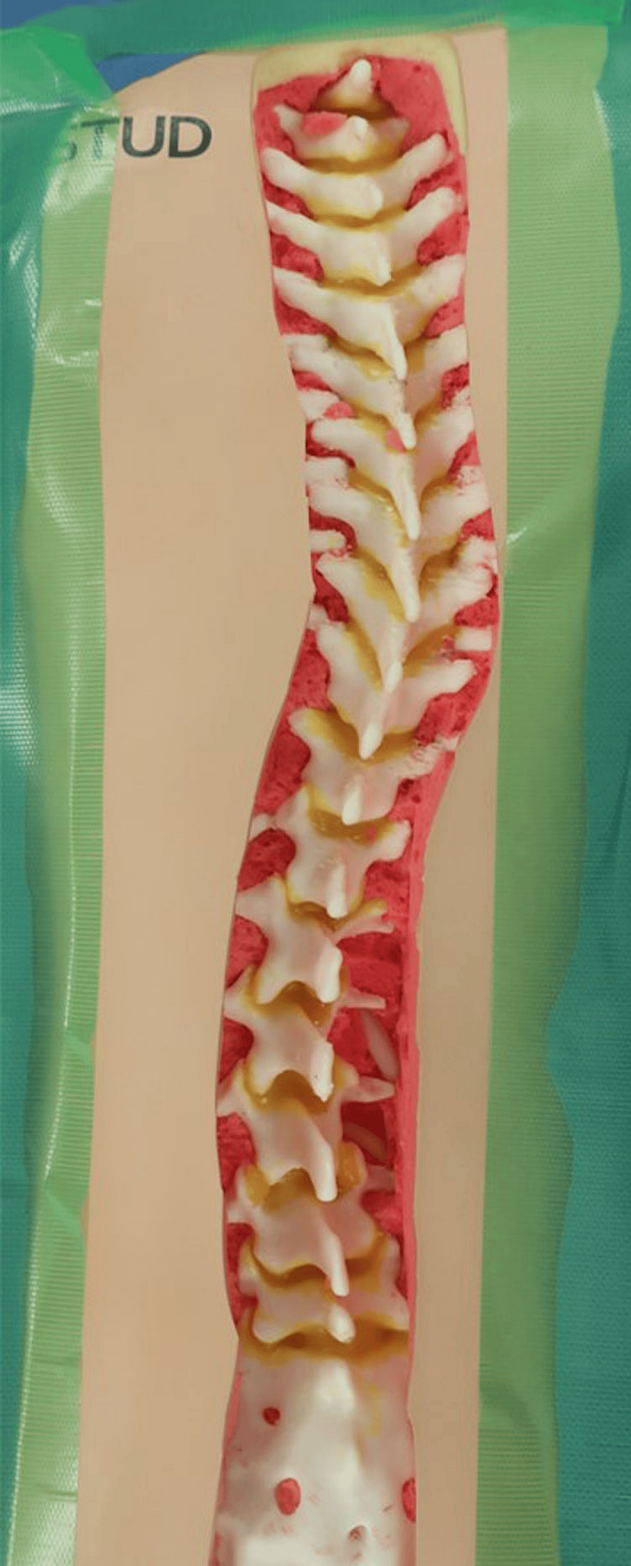
3D-printed spine model of the patient with adolescent idiopathic scoliosis

Using the preoperative CT-DICOM data of our patient, a 3D-printed spine model with a radiolucent soft tissue envelope was created (SurgiSTUD™, Tempe, AZ) (Figure 2) for the experiment of PS insertion under MR guidance. This kind of 3D-printed model has been already utilized for surgical training, including PS insertion [15].

**Figure 3:**
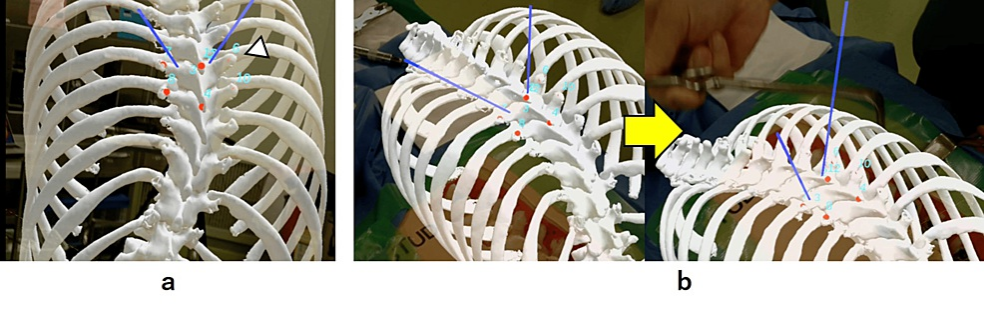
Registration of the 3D hologram via Magic Leap One headset a. Three markers (red points; tips of the spinous process and bilateral transverse processes) were set on the vertebra with interest (arrowhead). Additionally, several markers were set on the upper and lower vertebrae to improve the accuracy of superimposition of the 3D hologram onto the 3D-printed spine model. Trajectories for pedicle screws were also set (blue lines). b. The superimposition of the hologram onto the 3D spine model was performed manually by aligning the landmarks.

The preoperative CT-DICOM data of our patient was converted into stereolithography (STL) file format using the MIMICS® software (Materialise Japan Co., Ltd., Yokohama, Japan). To edit 3D triangular meshes, the STL file was imported into the MeshLab software (v2022.02, ISTI-CNR, Pisa, Italy) followed by importing into the Meshmixer design software program (Autodesk, Inc. San Rafael, CA). Finally, the 3D mesh data was uploaded to the Holoeyes MD service website (Holoeyes Inc. Tokyo, Japan), which automatically created MR data, which was referred to as a hologram. Furthermore, the data was downloaded to a commercial AR/MR device, a Magic Leap One (ML1) headset (Magic Leap Inc., Plantation, FL). Using the goggles of the headset, the surgeon could directly view the 3D-printed spine model as well as the 3D hologram. In addition, ML1 was equipped with a controller which allowed 3D movements of the hologram. For the registration via ML1 headset, the surgeon performed manual alignment of the hologram along three anatomic points for each vertebra (tips of the spinous process, transverse processes, or articular processes), which were marked on the 3D-printed spine model using a wireless remote control (Figure 3a). This planning procedure used six or more registration points on two or three consecutive vertebrae. The spine hologram was superimposed on the 3D spine model as seen through the goggles of the ML1 headset (Figure 3b). 

Blinded to the CT scan and X-ray images of the spine, a board-certified, experienced spine surgeon, who had had no experience with MR-assisted procedures before this experiment, performed bilateral PS insertion on each vertebra from T5 to L3 while wearing the ML1 headset, referencing the predetermined insertion points and trajectory lines of PS on the superimposed hologram. After preparation of the insertion points with a 3-mm perforating drill, a screw hole was created using a probe, the wall was scouted by sounding, and a tap hole was created. The wall was again scouted by sounding before inserting the PS. All procedures were performed along with the trajectory lines of PS on the superimposed hologram of the spine. The diameter and length of PSs used in the experiment were the same as those used in PSF for AIS. Due to the differences between positioning during surgery (prone position) and during preoperative CT, the manual alignment of the hologram and MR-assisted PS placement were performed for every two or three consecutive vertebrae. The duration between this experiment and the PSF was four months, which was a sufficient interval between the actual surgery and this experiment to eliminate the influence of memory during the actual surgery using CT-based navigation.

Control experiment

**Figure 4:**
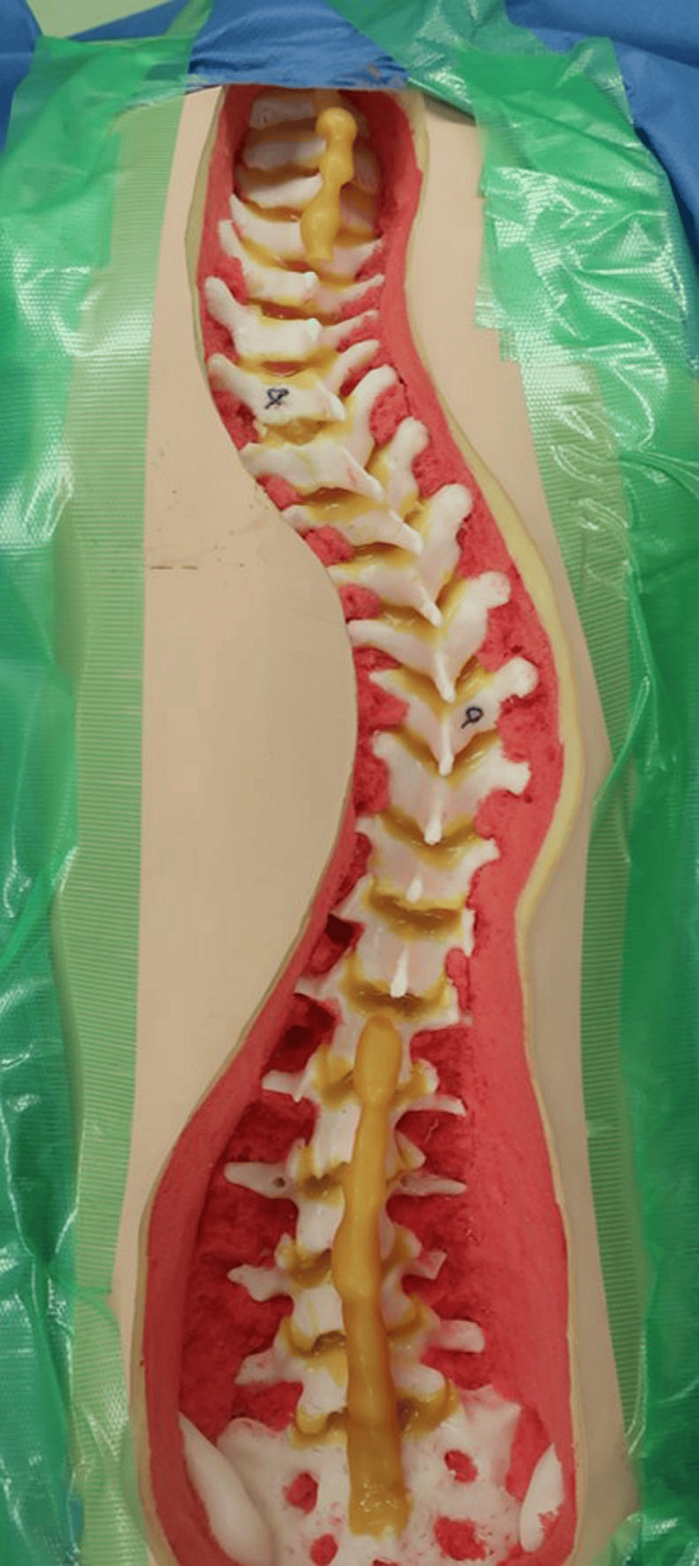
Readymade 3D spine model of adolescent idiopathic scoliosis created without any imaging data, including radiographs and computed tomography

Using a readymade 3D model of AIS (SurgiSTUD™, Tempe, AZ) (Figure 4), the same surgeon inserted PS from T4 to L3 with a freehand technique. The surgeon had no radiographic and CT data of the patient for this 3D model. In the freehand technique, a starting point for PS was determined based on anatomical landmarks such as the transverse process and superior articular process. Next, the pedicle probe was inserted, relying on the tactile feedback of the surgeon. A ball-ended feeler was used to ensure no breaches. After the tapping and probing were repeated, an appropriate-size screw was then inserted along the pedicle track.

Assessment of PS position

After inserting all PS into the 3D spine models, laminectomies and total facetectomies were performed. Then, all pedicle walls except lateral walls were visualized. Next, the 3D spine models were removed from the soft tissue envelope. Finally, it was determined for each vertebra whether the screw was contained within the pedicle or deviated (partially or completely) from the pedicle.

Statistical analysis

Statistical analyses were performed using SPSS software version 27 (IBM Corp., Armonk, NY). The accuracy of PS placement was compared between MR-assisted and freehand techniques using Fisher’s exact test. P-value <0.05 was considered statistically significant.

## Results

**Figure 5:**
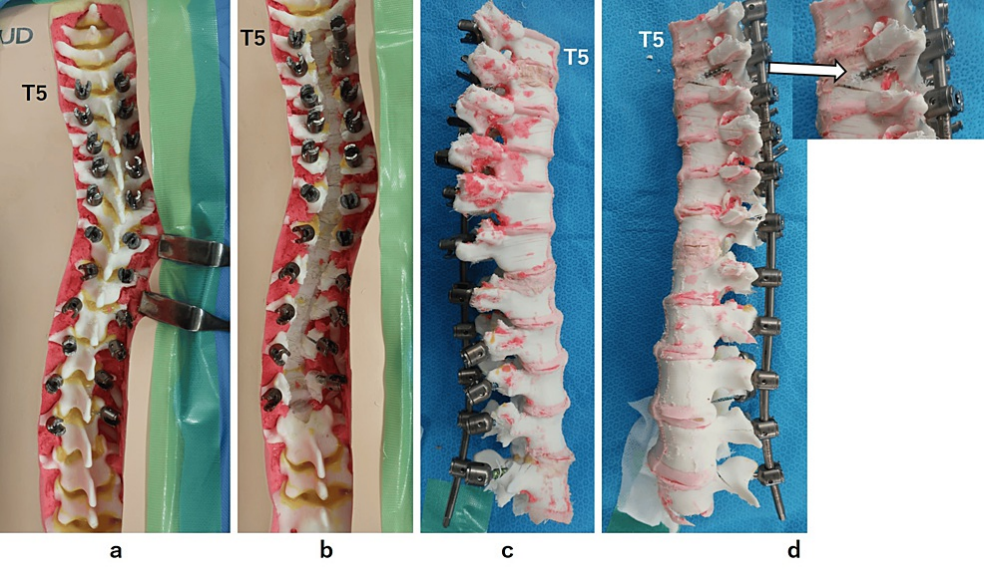
Post-experimental photographs of the 3D-printed spine model of the patient with adolescent idiopathic scoliosis a. Pedicle screws were placed bilaterally from T5 to L3 using a mixed reality-assisted technique. b. After laminectomy from T5 to L3, no medial pedicle violations were detected. c. Right lateral aspect of the vertebral column, where no lateral pedicle violations were detected. d. Left lateral aspect of the vertebral column, where the lateral breach of the pedicle screw was detected at T6.

**Figure 6:**
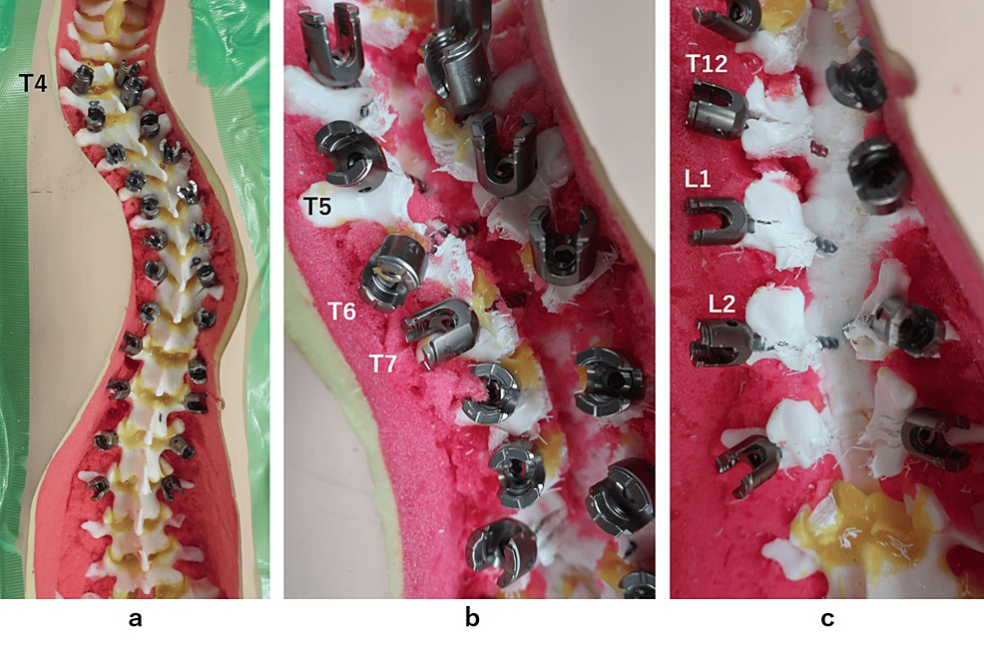
Post-experimental photographs of the readymade 3D spine model a. Pedicle screws were placed bilaterally from T4 to L3 with freehand technique. b. After laminectomy from T4 to T10, medial pedicle violations were detected at the left T5, 6, and 7. c. After laminectomy from T11 to L3, medial pedicle violations were detected at the left T12 and L1, and at bilateral L2.

In the 3D-printed spine model of our patient with AIS, 22 PSs were placed from T5 to L3 using the MR-assisted technique. Of the screws placed, only one PS deviated laterally (in-out-in trajectory) at left T6 (Figure 5). Therefore, a major breach occurred in 4.5% (1/22 screws). In a readymade 3D-printed spine model, 24 PSs were placed from T4 to L3 using the freehand technique. Seven of 24 screws (29.2%) demonstrated medial breach at left T5, 6, 7, 12, and L1, and at bilateral L2 (Figure 6), while there was no grossly evident lateral breach of the PS. The rate of PS malposition was significantly higher in the freehand technique than in the MR-assisted technique (p = 0.049).

## Discussion

The conventional technique of PS placement is a freehand approach using anatomical landmarks combined with or without 2D fluoroscopy guidance. However, rates of screw misplacement with ≥2 mm pedicle violation in the thoracolumbar spine range from 40% to 66% for freehand approach [1] and from 0% to 53% for that with 2D fluoroscopy guidance [1,2,5]. To improve the accuracy and safety of PS placement, various intraoperative navigation or guiding systems have been developed, including preoperative CT-based navigation, intraoperative CT- or 3D fluoroscopy-based navigation, and robot-assisted systems. In general, compared to the freehand technique, those systems significantly decreased the rate of PS malposition, which are 0% to 11% for preoperative CT-based navigation [1-3], 0% to 6.6% for 3D fluoroscopy based-navigation [2,3], 0% to 3.1% for intraoperative CT-based navigation [3,4], and 0% to 17.7% for robot-assisted systems [5,6].

More recently, new image processing technologies, including AR and MR, are developing for application in the field of spine surgery, especially PS placement. For AR-assisted techniques, a wide range of PS misplacements have been reported from 0% to 73.7%, while the majority of previous studies demonstrated clinically acceptable accuracy with a misplacement rate of <10% [14,16,17]. However, AR-assisted systems usually require intraoperative CT or 3D fluoroscopy [14,17,18], which is one of the barriers to the widespread adoption of AR technology for PS placement. The usual machines like intraoperative CT or fluoroscopy come at high costs, occupy large areas of space, make it difficult to maintain the sterility of the surgical field, and increase radiation exposure [19]. In contrast, MR technology can be integrated into existing operating rooms with low financial burden and clinical benefits. Wu et al. reported the feasibility of MR-based navigation for C1 lateral mass and C2 PSs using 3D-printed models [20]. In their experiments, no screw was misplaced outside the pedicle under MR-based navigation without radiation during screw insertion. Additionally, Li et al. reported accurate PS placement under MR-based navigation using 3D-printed models of lumbar fractures [21]. However, the models used in those experiments did not have soft tissue envelopes, which might allow the surgeon to directly visualize the lateral masses and pedicles.

In the current study, we evaluated the accuracy of MR-based navigation for PS placement using a 3D spine model of AIS with a soft tissue envelope, which provided a similar surgical field to the actual surgery. Moreover, we did not use fluoroscopy before and during PS placement. As a result, the rate of pedicle violation under MR-based navigation (4.5%) was significantly lower than those with freehand technique (29.2%). Because of the four-month interval between this experiment and the actual surgery, we believe that the accuracy of PS placement in the 3D-printed spine model of our patient was due to the MR-assisted navigation. We recognize that our method of MR-assisted PS placement can be adopted only for conventional open spine surgeries because the superimposition of the hologram onto the 3D-printed spine model used bony landmarks including spinous processes, transverse processes, and articular processes. For percutaneous PS placement in minimally invasive spine surgery, several researchers evaluated AR- or MR-based navigation systems that used superficial skin markers to superimpose holograms onto the patient, though the accuracy of PS placement in those systems was still inferior to conventional navigation systems [22,23]. However, the current rapid growth in the advancement of AR, VR, and MR technologies and their integration with other technologies such as artificial intelligence and machine learning will improve instrument guidance.

There are limitations in the current study. Firstly, PS placement was performed by a single, experienced spine surgeon. Therefore, further evaluations of various surgeons including fellows of orthopedic surgery and neurosurgery, are necessary. Secondly, the current experiment used only two spine models, which might provide different tactile feedback from the human vertebrae. Thirdly, the level of PS placement in our study did not include the cervical spine and lower lumbar spine. Fourthly, we did not have any data regarding the time needed for preparation of MR-based navigation and placement of PS because the current study is a preliminary study using 3D-printed spine models. Finally, we did not have control groups of PS placement under 2D fluoroscopy guidance or CT-based navigation systems. Therefore, we are planning a cadaveric study to evaluate the accuracy and feasibility of MR-assisted PS placement from cervical to lumbosacral spine compared to PS placement with freehand technique, 2D fluoroscopic guidance, and CT-based navigation systems. 

## Conclusions

This study demonstrated a significantly lower rate of PS misplacement in the MR-based technique than in the freehand technique. Additionally, our method of MR-based PS placement did not require intraoperative CT or 2D/3D fluoroscopy guidance. Therefore, the MR-assisted technique is a promising tool for PS placement without radiation in terms of feasibility, safety, accuracy, and cost-saving, warranting further studies including cadaveric and clinical studies.
